# High resolution mass spectral data from the analysis of copper chlorophylls and copper chlorophyll degradation products in bright green table olives

**DOI:** 10.1016/j.dib.2020.105548

**Published:** 2020-04-23

**Authors:** Peter F. Scholl, Patrick J. Gray, Bhakti Petigara Harp, Pierluigi Delmonte

**Affiliations:** Center for Food Safety and Applied Nutrition, U.S. Food and Drug Administration, 5001 Campus Drive, College Park, MD 20740

**Keywords:** Mass spectrometry, Food adulteration, Table olives, Chlorin, Copper chlorophylls

## Abstract

This publication reports high resolution mass spectral data for copper chlorophyll and copper chlorophyll degradation products extracted from bright green table olives. These data support analyte identifications made in “Quantitation of copper chlorophylls in green table olives by ultra-high-performance liquid chromatography with inductively coupled plasma isotope dilution mass spectrometry” in the *Journal of Chromatography* A (Petigara Harp *et al.*, 2020 [1]). Table olive pigments, divided into lipophilic and hydrophilic fractions by liquid-liquid repartition, were separated by ultra-high-performance liquid chromatography and detected by visible wavelength absorbance and high resolution mass spectrometry, using an Orbitrap HF with positive electrospray ionization. Full-scan mass spectra were acquired to assign pigment chemical formulae. Fragment-rich higher-energy collisional dissociation tandem mass spectra were acquired to facilitate structural assignments. Extracted ion chromatograms, full-scan, and tandem mass spectra obtained from representative lipophilic and hydrophilic green table olive extracts are presented in Figures 1-6. Annotated mass spectra comparing experimental and calculated isotope distributions, .raw mass spectral data files, and experimental details linking .raw data files to annotated spectra are provided as Supplementary Material. Spectra extracted from these native data files can be added to mass spectral libraries for use in other studies. Access to native data files uniquely enables rigorous data examination (*e.g.*, molecular ion isotopic distribution, effective mass resolution, presence of overlapping ion series) and use in ways that are not possible when spectra are otherwise reported in simple tables listing mono-isotopic peaks and mass errors. Mass spectra reported here can be used to design multiple-reaction monitoring methods to detect these bright green pigments in agricultural food commodities and finished products.

Specifications Table

**Table:** 

Subject	Food Science
Specific subject area	Analytical chemistry, chlorin mass spectrometry, table olive color adulteration
Type of data	Annotated MS1 and MS2 spectra, raw mass spectrometry data files
How data were acquired	Positive electrospray ionization UHPLC-MS1 and higher-energy collisional dissociation (HCD) tandem (MS2) mass spectrometry, Thermo Scientific QExactive HF mass spectrometer, Xcalibur Software (version 3.0.63)
Data format	Supplementary Material: Annotated MS1 and MS2 spectra: Microsoft Word .docx Thermo Scientific QExactive HF native mass spectral data: .raw files Raw mass spectral data file experimental parameters and their mapping to annotated spectra: Microsoft .xlsx
Parameters for data collection	Positive ionization electrospray UHPLC-MS1 and MS2 spectra were acquired. Mass resolving power settings ranged from 60,000-120,000 in MS1 studies. HCD spectra were acquired using different collision energies (10-130), mass resolving power settings (30,000-60,000), and parent molecular ion isolation widths (0.4 and 1.0 *m/z*). Individual .raw files and the Supplemental Material .xlsx file provide MS parameter details.
Description of data collection	UHPLC-MS1 and MS2 spectra were respectively acquired to assign chemical formulae and propose structures for copper chlorophylls and copper chlorophyll degradation products detected in lipophilic and hydrophilic bright green table olive extracts.
Data source location	College Park, Maryland, U.S.A.
Data accessibility	With the article
Related research article	Bhakti Petigara Harp, Peter F. Scholl, Patrick J. Gray, Pierluigi Delmonte, Quantitation of copper chlorophylls in green table olives by ultra-high-performance liquid chromatography with inductively coupled plasma isotope dilution mass spectrometry, *Journal of Chromatography A*, doi.org/10.1016/j.chroma.2020.461008.

## Value of the data

• MS1 data support the assignment of molecular formulae to copper chlorophylls and copper chlorophyll degradation products at <3 ppm mass error and provide free access to electrospray ionization spectra acquired at high (120,000) resolving power
• Fragment-rich MS2 data support proposed structural assignments for commercially unavailable compounds and provide some of the first electrospray positive ionization HCD spectra for copper chlorophylls and copper chlorophyll degradation products
• Raw mass spectrum data files enable chemists to independently evaluate, interpret, and include them in spectral libraries
• Data can be further used to design multiple reaction monitoring MS experiments for the targeted detection of green pigments in table olives and other food products
• Dataset may also be of use in geochemical studies of copper chlorophylls, chlorophyllins, chlorins, and related metallo-porphyrins

## 1 Data Description

**Fig. 1:**
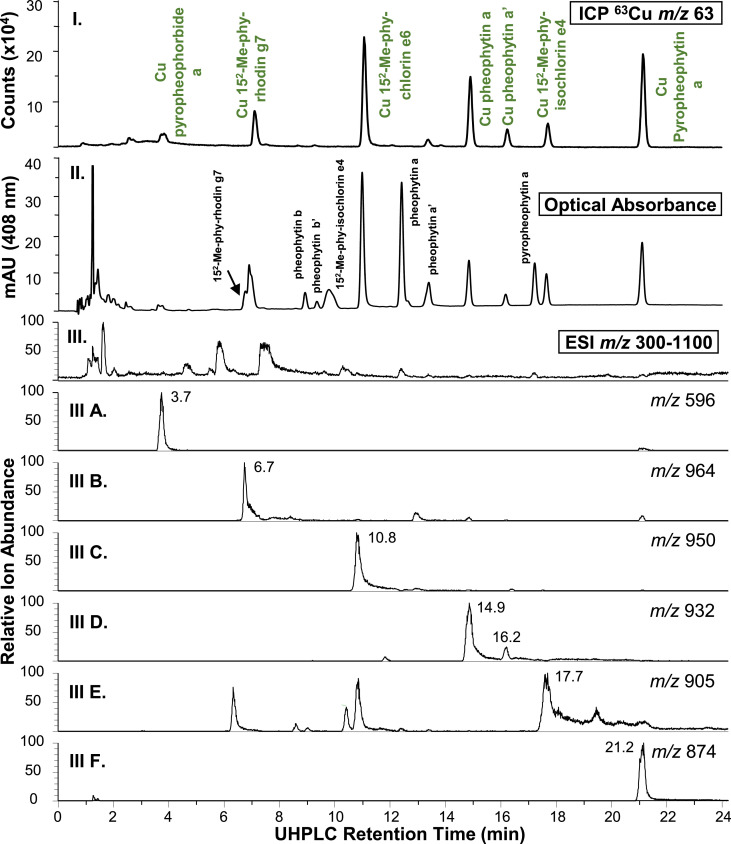
UHPLC analysis of lipophilic extract from bright green olives. (I.) ICP-MS 63Cu ion chromatogram; (II.) visible wavelength extracted (408 nm) chromatogram; (III.) MS1 total ion chromatogram (TIC, m/z 300-1100, 100% = 9.6E6); (A.) Cu pyropheophorbide a extracted ion chromatogram (XIC, (M+H)+, m/z 596.1700-596.1900, 100% = 5.4E4); (B.) Cu 152-Me-phytyl rhodin g7 ((M+H)+, m/z 964.3700-964.5700, 100% = 7.4E4); (C.) Cu 152-Me-phytyl chlorin e6 ((M+H)+, m/z 950.3970-950.5970, 100% = 1.9E5); (D.) Cu pheophytin a and a’ ((M+H)+, m/z 932.3873-932.5873, 100% = 1.2E5); (E.) Cu 152-Me-phytyl isochlorin e4 ((M.+, m/z 905.3998-905.5998, 100% = 8.1E4); (F.) Cu pyropheophytin a ((M+H)+, m/z 874.3809-874.5809, 100% = 2.3E5). Although not co-collected, ICP-MS and MS1 XICs are co-presented to facilitate comparison to Fig. 2 reported by Petigara *et al*. (2020) and support chemical formulae assignments.[1] Raw MS Data file: C(L).raw.

**Fig. 2:**
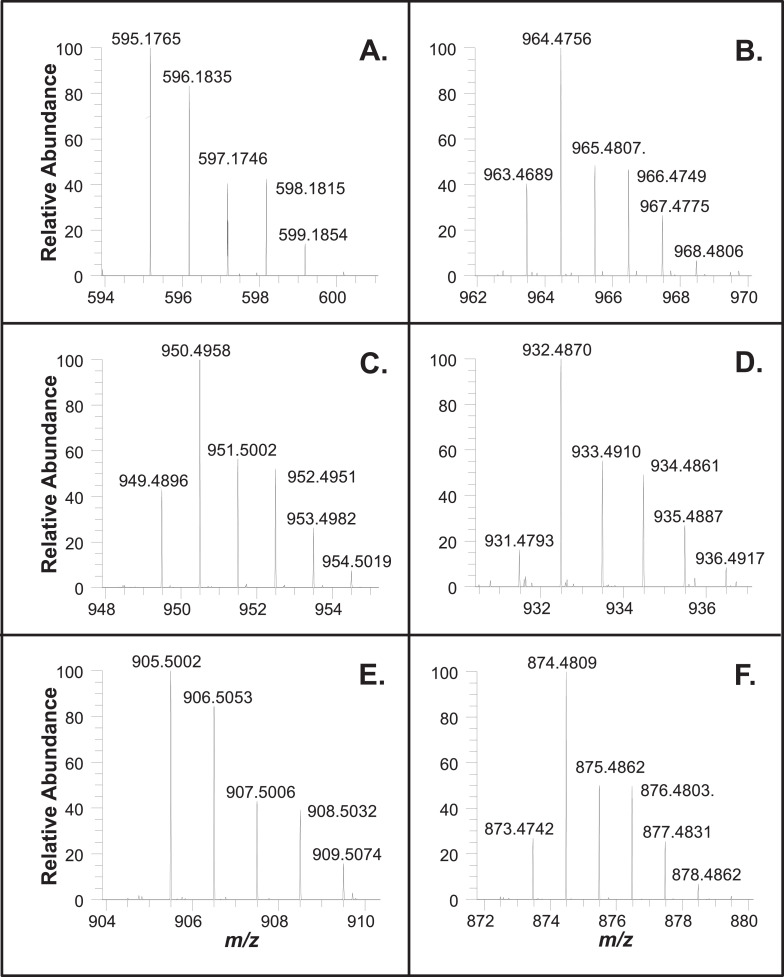
MS1 spectra of chromatographically separated copper chlorophylls shown in Fig. 1. (A.) Cu pyropheophorbide a, 3.7 min (C33H32N4O3Cu, M.+ m/z 595.1765 (0.0 ppm mass error), (M+H)+ m/z 596.1835 (-1.3 ppm), 100% = 7.0E4); (B.) Cu 152-Me-phytyl rhodin g7, 6.7 min (C55H72N4O7Cu, M.+ m/z 963.4689 (-0.3 ppm), (M+H)+ m/z 964.4756 (-1.5 ppm), 100% = 5.2E4); (C.) Cu 152-Me-phytyl chlorin e6, 10.8 min (C55H74N4O6Cu, M.+ m/z 949.4896 (-0.3 ppm), (M+H)+ m/z 950.4958 (-2.0 ppm), 100% = 1.4E5); (D.) Cu pheophytin a, 14.9 min (C55H72N4O5Cu, M.+ m/z 931.4793 (0.0 ppm), (M+H)+ m/z 932.4870 (-0.1 ppm), 100% = 9.9E4); not shown: Cu pheophytin a’, 16.2 min (M.+ m/z 931.4796 (0.3 ppm), (M+H)+ m/z 932.4868 (-0.3 ppm)); (E.) Cu 152-Me-phytyl isochlorin e4, 17.6 min (C54H74N4O4Cu, M.+ m/z 905.5002 (0.1 ppm), (M+H)+ m/z 906.5053 (-2.9 ppm), 100% = 6.1E4); (F.) Cu pyropheophytin a, 21.2 min (C53H70N4O3Cu, M.+ m/z 873.4742 (0.5 ppm), (M+H)+ m/z 874.4809 (-0.9 ppm), 100% = 1.6E5). MS1 m/z 300-1100; resolving power 120,000. Calculated spectra for odd and even electron molecular ions are provided as Supplementary material. These data support chemical formula assignments made for chromatographic peaks (VIS and ICP-MS) reported in Table 1A and Fig. 2 of the work by Petigara *et al*. (2020).[1] Raw MS Data file: C(L).raw.

**Fig. 3:**
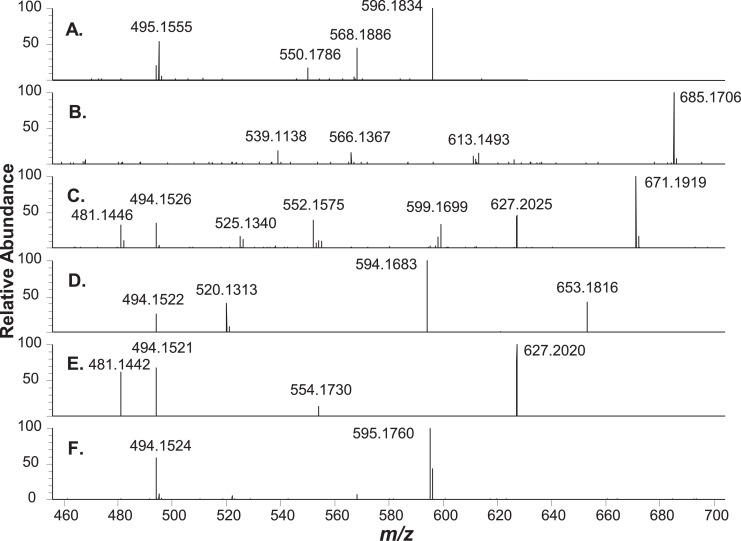
MS2 (HCD) spectra of chromatographically separated copper chlorophylls shown in Fig. 2. (A.) Cu pyropheophorbide a, 3.7 min (parent molecular ion (M+H)+ m/z 596.1832, 100% = 2.6E4); (B.) Cu 152-Me-phytyl rhodin g7, 6.7 min ((M+H)+ m/z 964.4756, 100% = 1.2E4); (C.) Cu 152-Me-phytyl chlorin e6, 10.8 min ((M+H)+ m/z 950.4975, 100% = 4.3E4); (D.) Cu pheophytin a, 14.9 min ((M+H)+ m/z 932.4873, 100% = 4.0E4); (E.) Cu 152-Me-phytyl isochlorin e4, 17.6 min ((M+H)+ m/z 906.5047, 100% = 3.6E4); (F.) Cu pyropheophytin a, 21.2 min ((M+H)+ m/z 874.4809, 100% = 5.3E4). Isolation width 0.4 m/z; collision energy spread 30, 35, 40; resolving power 60,000. HCD spectra for these and other compounds are presented as Supplementary material. Raw MS Data file: C(L).raw.

**Fig. 4:**
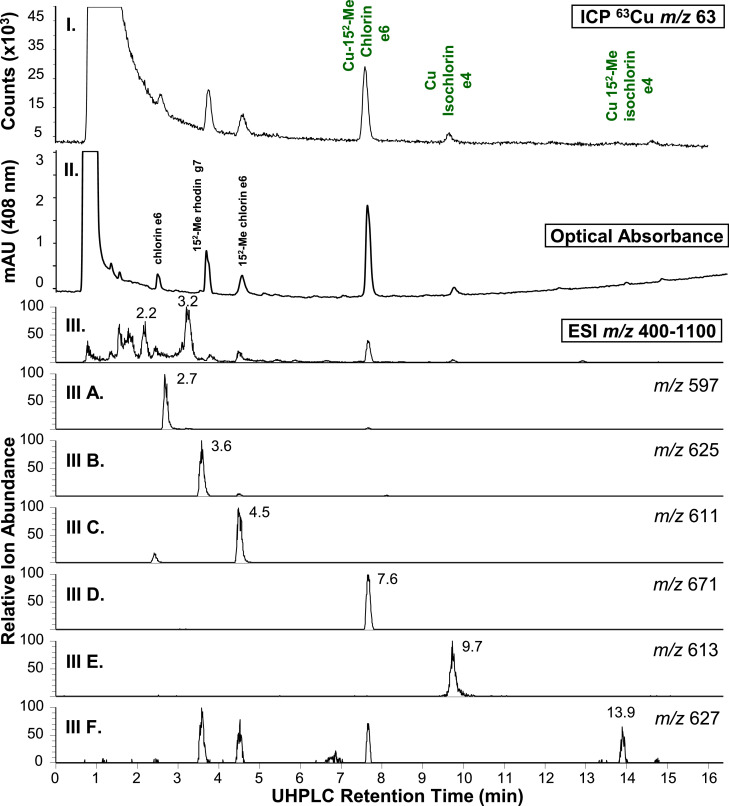
UHPLC analysis of hydrophilic extract from bright green olives. (I.) ICP-MS 63Cu ion chromatogram; (II.) visible wavelength extracted (408 nm) chromatogram; (III.) MS1 TIC (m/z 400-1100); (A.) chlorin e6 XIC ((M+H)+ m/z 597.2600-597.2800, 100% = 1.1E5); (B.) 152-Me rhodin g7 ((M+H)+ m/z 625.2500-625.2800, 100% = 8.6E4); (C.) 152-Me chlorin e6 ((M+H)+ m/z 611.2750-611.2950, 100% = 3.0E5); (D.) Cu 152-Me chlorin e6 ((M+H)+ m/z 672.1870-672.2070, 100% = 6.4E4); (E.) Cu isochlorin e4 ((M+H)+ m/z 614.1800-614.2000, 100% = 2.2E4); (F.) Cu 152-Me chlorin e4 ((M+H)+ m/z 628.1000-628.3000, 100% = 7.2E3). Although not co-collected, ICP-MS and MS1 XICs are co-presented to facilitate comparison to Fig. 3 reported by Petigara *et al*. (2020) and support chemical formulae assignments.[1] Raw MS Data file: B(H).raw.

**Fig. 5:**
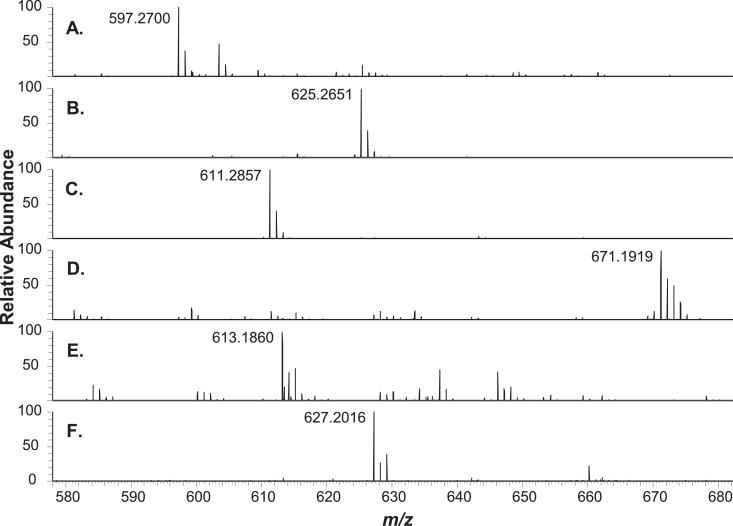
MS1 spectra of chromatographically separated copper chlorophyllins shown in Fig. 4. (A.) chlorin e6, 2.7 min (C34H36N4O6+H, (M+H)+ m/z 597.2700 (-1.3 ppm), 100% = 6.1E4); (B.) 152-Me rhodin g7, 3.6 min (C35H36N4O7+H, (M+H)+ m/z 625.2651 (-0.9 ppm), 100% = 5.3E4); (C.) 152-Me chlorin e6, 4.5 min (C35H38N4O6+H, (M+H)+ m/z 611.2857 (-1.1 ppm), 100% = 1.8E5); (D.) Cu 152-Me chlorin e6, 7.6 min (C35H36N4O6Cu, M.+ m/z 671.1919 (-0.9 ppm), 100% = 4.8E4); (E.) Cu isochlorin e4, 9.7 min (C33H34N4O4Cu, M.+ m/z 613.1860 (-1.8 ppm), 100% = 1.2E4); (F.) Cu 152-Me chlorin e4, 13.9 min (C34H36N4O4Cu, M.+ m/z 627.2016 (-1.8 ppm), 100% = 2.7E3). MS1 scan m/z 400-1100, resolving power 60,000. Calculated spectra for odd and even electron molecular ions are provided as Supplementary material. These data support chemical formula assignments made for chromatographic peaks (VIS and ICP-MS) reported in Table 1B and Fig. 3 of the work by Petigara *et al*. (2020).[1] Raw MS Data file: B(H).raw.

**Fig. 6:**
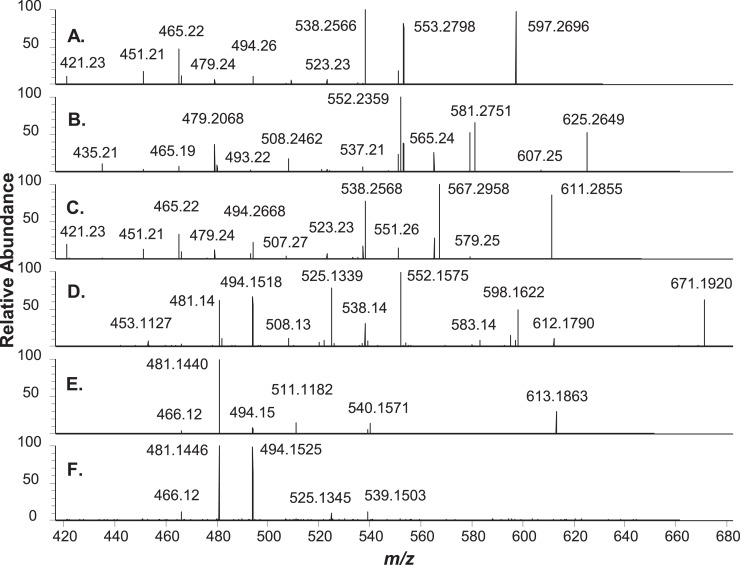
MS2 (HCD) spectra of chromatographically separated copper chlorophyllins shown in Fig. 5. (A.) chlorin e6, 2.7 min (parent molecular ion (M+H)+ m/z 597.2700, 100% = 4.7E4); (B.) 152-Me rhodin g7, 3.6 min ((M+H)+ m/z 625.2651, 100% = 2.8E4); (C.) 152-Me chlorin e6, 4.5 min ((M+H)+ m/z 611.2857, 100% = 1.1E5); (D.) Cu 152-Me chlorin e6, 7.6 min (M.+ m/z 671.1919, 100% = 2.6E4); (E.) Cu isochlorin e4, 9.7 min (M.+ m/z 613.1860, 100% = 4.8E4); (F.) Cu 152-Me chlorin e4, 13.9 min (M.+ m/z 627.2016, 100% = 7.7E3). Labels (m/z) were rounded down to accommodate space limitations. HCD spectra for these and other compounds are presented as Supplementary material. For A-D, E.: Isolation width 0.4 m/z; collision energy spread 30, 35, 40; resolving power 60,000. For F.: Isolation width 0.4 m/z; collision energy spread 35, 40, 45; resolving power 240,000. Raw MS Data files: (A-D.) C(H).raw; (E.) D(H).raw; (F.) G(H).raw.

High resolution full scan (MS1) and higher-energy collisional dissociation tandem (HCD-MS2) mass spectra were acquired using an Orbitrap HF spectrometer to assign chemical formulae and structures to lipophilic copper chlorophylls and hydrophilic copper chlorophyll degradation products extracted from bright green table olives. These data support chemical formulae assignments made for pigment analytes in reference [1]. UHPLC separation of copper chlorophylls extracted from a bright green table olive sample using Cu isotope inductively coupled plasma (Cu ICP) MS, optical absorbance, and positive electrospray ionization MS1 detection is presented in Figure 1. MS1 and HCD-MS2 spectra acquired at retention times corresponding to lipophilic analyte peaks in Fig. 1. are respectively presented in Fig. 2, Fig. 3. Similarly to Fig. 1, Fig. 4 presents the UHPLC separation of copper chlorophyll degradation products extracted from a bright green table olive sample. Fig. 5, Fig. 6 respectively present MS1 and HCD-MS2 spectra acquired at retention times corresponding to hydrophilic analytes in Fig. 4. Annotated MS1 spectra, annotated HCD-MS2 spectra, and raw mass spectral data files are provided as Supplementary Material. Also, experimental details related to raw data files and further details linking raw data files to both Fig. 1, Fig. 2, Fig. 3, Fig. 4, Fig. 5, Fig. 6 and annotated spectra are provided in the Supplementary Material. Experimental MS1 spectra are annotated by presentation with calculated spectra for co-detected even and odd-electron molecular ions. References are provided for specialists that seek details regarding fragmentation mechanisms and the interpretation of metallo-chlorin mass spectra that are important to this work.[2], [3], [4], [5], [6], [7], [8], [9], [10], [11], [12], [13], [14], [15], [16]

## 2 Experimental Design, Materials, and Methods

### 2.1 UHPLC-UV-Vis analyses of table olive extracts

Lipophilic and hydrophilic table olive extracts prepared as described in reference [1] were analyzed using a Waters Acquity UPLC system (Waters Corp, Milford, MA) equipped with an Acquity PDA eλ detector and Ascentis Express C18 (2.1 × 150 mm, 2 µm, Millipore Sigma, Bellefonte, PA) column. All chromatograms were acquired from 300-800 nm at 0.35 mL/min, but different gradients and injection volumes were used for each type of extract.

Lipophilic extracts were separated with an elution gradient of (A) 0.1% acetic acid in methanol and (B) 50:50 acetonitrile/acetone as follows: from 0-100 % B in 25 min, hold for 5 min at 100% B, from 100-0 % B in 0.1 min, and 10 min of re-equilibration. The injection volume was 5 μL and the elution temperature was 27°C.

Hydrophilic extracts were separated with an elution gradient of (A) 0.1% formic acid in water and (B) acetonitrile as follows: from 35-100 % B in 20 min, hold for 5 min at 100% B, from 100-35 % B in 0.1 min, and 10 min of re-equilibration. The injection volume was 10 μL and the elution temperature was 30°C.

### 2.2 UHPLC-HRMS1 and higher-energy collision dissociation MS2 analyses

The UHPLC-PDA system was connected to a QExactive HF Orbitrap mass spectrometer (ThermoFisher Scientific, Bremen, Germany). Positive ESI MS1 and HCD spectra were acquired at mass resolving powers varying from 30 to 240k. Heated ionization source parameters were tuned via post-column infusion (125 μL/min) of sodium copper chlorophyllin in 50:50:0.1 acetonitrile/water/trifluoroacetic acid to: V_s_ 4 kV; capillary 300°C; probe heater 438°C; S-lens 55; sheath gas 53; and auxiliary gas 38. External mass calibration provided a mass error tolerance of <3 ppm. MS1 and HCD spectra were acquired during the post-column infusion (120 μL/min) of 0.1% formic acid in 50:50 acetonitrile/water for lipophilic extract and 1% acetic acid in 50:50 acetonitrile/water for hydrophilic extract. HCD spectra were obtained using N_2_ collision gas, stepped normalized collision energy (30-45) with a data-dependent or parallel reaction monitoring method, and variable parent molecular ion isolation width (0.4 or 1.0 *m/z*). Detailed instrument parameters are provided as Supplementary Material and within individual .raw data files. A variety of instrumental parameter (*e.g.*, resolution, collision energy) ranges and data acquisition techniques (*e.g.*, MS1 only, top 10 MS2, and unscheduled and scheduled MS2) were used. In one case, the green table olive lipophilic extract UPLC peak eluting at 12.3 min (Cu 15^2^-Me-phytyl-isorhodin g5) was hand collected and reanalysed via direct infusion (10 μL/min) for MS1 (*m/z* 300-2000) analysis and the separate HCD analysis of the odd (*m/z* 919.50) and even (*m/z* 920.50) parent molecular ions at collision energies ranging from 10-130.

## Conflict of Interest

The authors declare no competing financial interests or personal relationships which have, or could be perceived to have, influenced the work reported in this article.

## Appendix Supplementary materials

Image, application 1
