# Supplementary material for: High resolution mass spectral data from the analysis of copper chlorophylls and copper chlorophyll degradation products in bright green table olives
Source: Data Brief. 2020 Apr 23;30:105548. doi: 10.1016/j.dib.2020.105548 (PMC7200247; doi:10.1016/j.dib.2020.105548)
Supplement: Supplementary file 1 [file mmc1.zip › R2_Supplementary Material/R2_Supplementary_Material_Mass_Spectra.docx]

**Supplemental Materials:**

**Scholl *et. al*, “**High resolution mass spectral data from the analysis of copper chlorophylls and copper chlorophyll degradation products in bright green table olives**” *Data in Brief*, 2020.**

**MS1 and MS2 (HCD) Spectra**

1. **Lipophilic Compounds**

Figure # Name Page #

1.0 Cu pheophorbide a 2

2.0-2.1 Cu pyropheophorbide a 3-4

3.0-3.1 15^2^-Me-phytyl rhodin g7 5-6

4.0-4.1 Cu 15^2^-Me-phytyl rhodin g7 7-8

5.0-5.1 Pheophytin b and b’ 9-10

6.0-6.1 Cu Pheophytin b and b’ 11-12

7.0-7.1 15^2^-Me-phytyl isochlorin e4 13-14

8.0-8.2 Cu 15^2^-Me-phytyl chlorin e6 15-17

9.0-9.1 Cu 15^2^-Me-phytyl isorhodin g5 18-19

10.0-10.1 Pheophytin a and a’ 20-21

11.0-11.2 Cu Pheophytin a and a’ 22-24

12.0-12.1 Pyropheophytin a 25-26

13.0-13.1 Cu 15^2^-Me-phytyl isochlorin e4 27-28

14.0-14.1 Cu pyropheophytin a 29-30

1. **Hydrophilic Compounds**

Figure # Name Page #

15.0-15.1 Chlorin e6 31-32

16.0-16.1 15^2^-Me rhodin g7 33-34

17.0-17.1 15^2^-Me chlorin e6 35-36

18.0-18.1 15^2^-Me isochlorin e4 37-38

19.0-19.1 Cu 15^2^-Me chlorin e6 39-40

20.0-20.3 Cu isochlorin e4 41-44

21.0-21.1 Pyropheophorbide a 45-46

22.0-22.1 Cu 15^2^-Me isochlorin e4 47-48

1. **References 49**

**A. MS1 and HCD Spectra: Lipophilic compounds**


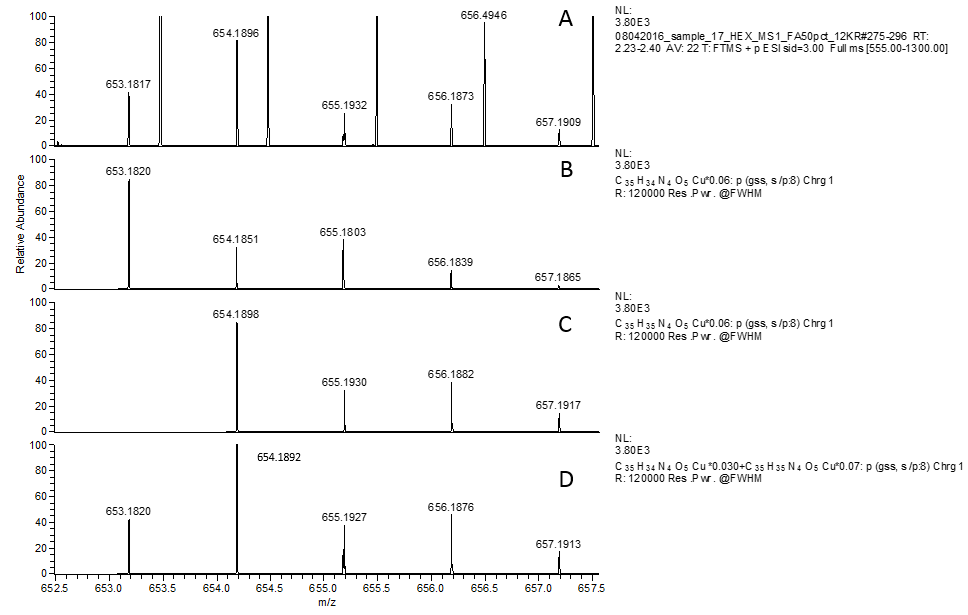


**Figure 1.0. MS1 spectrum of Cu pheophorbide a (C35H34N4O5Cu) co-detected with unidentified coeluting material in lipophilic olive extract.** Experimental MS1 spectra (**A**) at T_r_ = 2.4 min were complex; they exhibited a mixture of ions containing Cu pheophorbide a M^+.^, (M+H)^+^ and unidentified co-eluting interferences. The corresponding chromatographic absorbance (408 nm) peak was very weak and this early eluting region exhibited a high ^63^Cu background ICP-MS signal. Attempts to acquire confirmatory HCD spectra of Cu pheophorbide a failed. **(A)**: experimental spectra (resolution (R) = 120k) exhibited weak intensity Cu pheophorbide a M^+.^ (*m/z* 653.1817) and (M+H)^+^ (*m/z* 654.1896) ions; (**B**) calculated spectrum for Cu pheophorbide a M^+.^; (**C**) calculated spectrum for Cu pheophorbide a (M+H)^+^; (**D**) calculated spectrum for a mixture (1.0:2.3) of Cu pheophorbide a M^+.^ and (M+H)^+^. Experimental mass errors, respectively calculated for M^+.^ and (M+H)^+^ using monoisotopic ions in panels A and D, were 0.5 and -0.8 ppm. See data file A(L).raw for details.


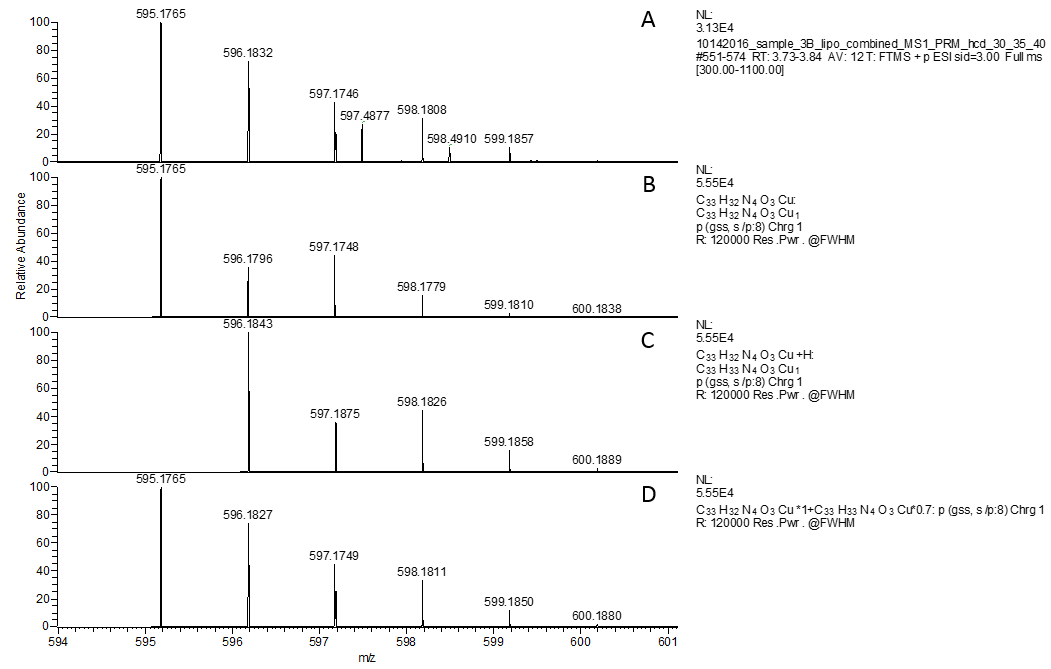
**Figure 2.0. MS1 spectra of Cu pyropheophorbide a (C33H32N4O3Cu ) in lipophilic olive extract.** Overlapping Cu pyropheophorbide a M^+.^ and (M+H)^+^ isotopic envelopes (Tr = 3.7 min) distort the experimental spectrum (**A**) to mask the expected signature isotopic distribution indicating the presence of copper, seen in the calculated Cu pyropheophorbide a spectra of these individual odd (**B**) and even (**C**) electron ions. However, the calculated spectrum (**D**) of a (1.0:0.7) mixture of Cu pyropheophorbide a M^+.^ and (M+H)^+^ respectively matches the experimental spectrum to 0.0 and -0.8 ppm mass error. (**A**): experimental spectrum at T_r_ = 3.7 min (R = 120k) exhibiting M^+^ (*m/z* 595.1765) and (M+H)^+^ ions (*m/z* 596.1832); (**B**): calculated M^+.^ spectrum; (**C**): calculated (M+H)^+^spectrum; (**D**) calculated spectrum of a (1.0:0.7) mixture of M^+.^ and (M+H)^+^. See data file B(L).raw for details.


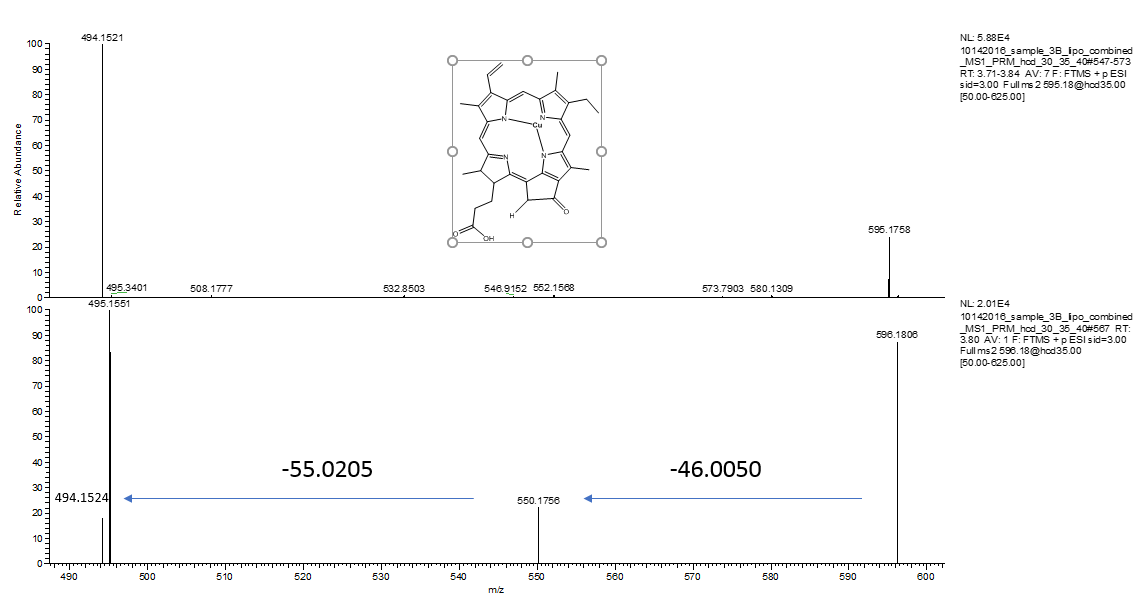


**Figure 2.1. HCD spectra of Cu pyropheophorbide a (C33H32N4O3Cu) in lipophilic olive extract** (T_r_ = 3.7 min). **Top panel:** M^+.^ (*m/z* 595.18); B**ottom panel**: (M+H)^+^ (*m/z* 596.18). Q1 = 0.4 Th; R = 60k; CE 30_35_40. Detection of Cu pyropheophorbide a was previously reported on the basis of observing M^+.^ in sodium copper chlorophyllin and (M+H)^+^ in olive and tea extracts; however, tandem mass spectra of the corresponding parent molecular ions have not been reported.^1-3^ SCIFINDER searching of the chemical formula (C33H32N4O3Cu) does not yield Cu-pyropheophorbide. For these reasons, the structure of Cu pyropheophorbide a has not been fully established. Comparison of this HCD spectrum to that of pyropheophorbide a is helpful in discerning the effect of Cu-coordination on fragmentation patterns. Shioi *et al.* 1996 reported the pyropheophorbide a positive ionization FAB spectrum exhibited a molecular ion peak at *m/z* 535 but did not specify what fragment ions were detected.^4^ See data files B(L).raw and C(L).raw for details.

**Figure 3.0.** MS1 spectrum observed: **Top panel:** 15^2^-Me-phytyl-rhodin g7, T_r_ 6.3 min, (C55H74N4O7), (M+H)^+^ *m/z* 903.5631**; Bottom panel:** Calculated spectra for (M+H)^+^. R = 120k. See data file B(L).raw for details.

**Figure 3.1. HCD spectrum of 15^2^-Me-phytyl-rhodin g7** ((M+H)^+^ *m/z* 903.56); Q1 = 0.4 Th; R = 60k; CE 30_35_40. See data file B(L).raw for details.

**Figure 4.0. MS1 spectrum observed (Top) of Cu 15^2^-Me-phytyl rhodin g7** (M^+.^ *m/z* 963.4692; (M+H)^+^ *m/z* 964.4770); R = 120 k. **Middle:** calculated for M^+.^; **Bottom:** calculated for (M+H)^+^. See data file C(L).raw for details.

**Figure 4.1. Targeted HCD spectrum of Cu 15^2^-Me-phytyl rhodin g7** ((M+H)^+^ *m/z* 964.48); Q1 = 0.4 Th; R = 60k; CE 30_35_40. See data file C(L).raw for details.

**Figure 5.0. MS1 spectra observed for (Top) Pheophytin b and (Middle) b’**((M+H)^+^ *m/z* 885.5525); R = 60k**. (Bottom):** calculated for (M+H)^+^. See data file D(L).raw for details.

**Figure 5.1. Targeted HCD spectra of Pheophytin b and b’** ((M+H)^+^ *m/z* 885.55); Q1 = 1.0 Th; R = 60k; CE 25_30_35. See data file E(L).raw for details.

**Figure 6.0. MS1 spectra observed for (Top) Cu Pheophytin b and (Middle) b’**((M+H)^+^ *m/z* 946.47); R = 60k. **(Bottom)** calculated for (M+H)^+^. See data file D(L).raw for details.

**Figure 6.1. HCD spectra of (Top) Cu Pheophytin b and (Bottom) b’** ((M+H)^+^ *m/z* 946.47); Q1 = 1.0 Th; R = 60k; CE 25_30_35. See data file E(L).raw for details.

**Figure 7.0. MS1 spectrum observed (Top) of 15^2^-Me-phytyl isochlorin e4** exhibiting M^+.^ *m/z* 844.5862 and (M+H)^+^ *m/z* 845.5940; **Middle**: calculated for M^+.^; **Bottom**: calculated for (M+H)^+^. R = 120K. See data file B(L).raw for details.

**Figure 7.1. Targeted HCD spectrum of 15^2^-Me-phytyl isochlorin e4** (M+H)^+^ *m/z* 845.59; Q1 = 0.4 Th; R = 60k; CE _30_35_40. See data file B(L).raw for details.

**Figure 8.0. MS1 spectrum of Cu 15^2^-Me-phytyl chlorin e6 (Top)** exhibiting M^+.^ *m/z* 949.4906 and (M+H)^+^ *m/z* 950.4975); R = 60K. **Middle**: calculated for M^+.^; **Bottom**: calculated for (M+H)^+^. See data file D(L).raw for details.

******Figure 8.1. Targeted HCD spectra of Cu 15^2^-Me-phytyl chlorin e6, Top:** M^+.^ *m/z* 949.49; **Bottom:** (M+H)^+^ *m/z* 950.50); Q1 = 1.0 Th; R = 60k; CE 25_30_35. See HCD spectrum close-up on the next page. See data file E(L).raw for details.

**Figure 8.2. Close-up of HCD spectra of Cu 15^2^-Me-phytyl chlorin e6, Top:** M^+.^ *m/z* 949.49; **Bottom:** (M+H)^+^ *m/z* 950.50); Q1 = 1.0 Th; R = 60k; CE 25_30_35. See data file E(L).raw for details.

**
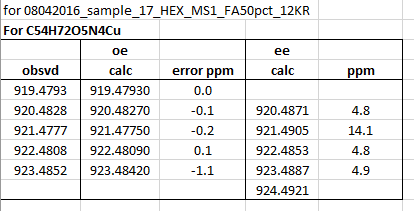
**

**Figure 9.0. MS1 spectra observed (Top panel) of proposed Cu 15^2^-Me-phytyl isorhodin g5; Middle:** calculated M^+.^; **Bottom**: calculated (M+H)^+^ spectra. R = 120,000. Infused, hand collected fraction. See data file A(L).raw for details.

**Figure 9.1. HCD spectra of (M+H)^+^ (Top) and M^+.^ (Bottom) for Cu 15^2^-Me-phytyl isorhodin g5.** Infusion. See data file F(L).raw for details.

**Figure 10.0. MS1 spectra of Pheophytin a (Top) and a’ (Middle) ((M+H)^+^ *m/z* 871.57)**; R = 60k. **Bottom**: calculated for (M+H)^+^. See data file D(L).raw for details.

**Figure 10.1. Targeted HCD spectra of Pheophytin a (Top) and a’ (Bottom) ((M+H)^+^ *m/z* 871.57)**; Q1 = 1.0 Th; R = 60k; CE 25_30_35. See data file E(L).raw for details.

**Figure 11.0. MS1 spectra of Cu Pheophytin a (Top) and a’ (Second from top)** ((M+H)^+^ *m/z* 932.49); R = 60k. (**Third from top**): calculated for M^+.^; **Bottom**: calculated for (M+H)^+^. See data file D(L).raw for details.

**Figure 11.1. Targeted HCD spectra of Cu Pheophytin a (Top) and a’ (Bottom)** ((M+H)^+^ *m/z* 932.49); Q1 = 1.0 Th; R = 60k; CE 25_30_35. See data file E(L).raw for details.

**Figure 11.2. Targeted HCD spectra of Cu Pheophytin a (Top) and a’ (Bottom)** ((M+H)^+^ *m/z* 932.49); Q1 = 1.0 Th; R = 60k; CE 25_30_35. See data file E(L).raw for details.

**Figure 12.0. MS1 spectrum observed (Top) of Pyropheophytin a** ((M+H)^+^ *m/z* 813.57); R = 120k. **Bottom:** calculated for (M+H)^+^. See data file B(L).raw for details.

**Figure 12.1. Targeted HCD spectrum of Pyropheophytin a** ((M+H)^+^ *m/z* 813.57); Q1 = 0.4 Th; R = 60k; CE 30_35_40. See data file B(L).raw for details.

******Figure 13.0. MS1 spectrum observed (Top) of Cu-15^2^-Me-phytyl isochlorin e4** (M^+.^ *m/z* 905.50); R = 120k. **Bottom**: calculated for M^+.^. See data file B(L).raw for details.

**Figure 13.1. Targeted HCD spectrum of Cu-15^2^-Me-phytyl isochlorin e4** (M^+.^ *m/z* 905.50); Q1 = 0.4 Th; R = 60k; CE 30_35_40. See data file B(L).raw for details.

**Figure 14.0. MS1 spectrum observed (Top) of Cu pyropheophytin a** (M+H)^+^ *m/z* 874.48); R = 120k; **Middle**: calculated for M^.+^; **Bottom**: calculated for (M+H)^+^ . See data file B(L).raw for details.

**Figure 14.1. Targeted HCD spectra of Cu pyropheophytin a: Top:** (M^+.^ *m/z* 873.47); **Bottom:** (M+H)^+^ *m/z* 874.48; Q1 = 0.4 Th; R = 60k; CE 30_35_40. See data file B(L).raw for details.

**B. MS1 and HCD Spectra: Hydrophilic Compounds**

******Figure 15.0. MS1 spectrum observed (Top) of chlorin e6** ((M+H)^+^ *m/z* 597.2706); R = 120k. **Middle**: calculated M^+.^; **Bottom**: calculated (M+H)^+^.See data file A(H).raw for details.

******Figure 15.1. Targeted HCD spectrum of chlorin e6** ((M+H)^+^ *m/z* 597.27); Q1 = 0.4 Th; R = 60k; CE 30_35_40. Compare to Isakau HA *et al*., *J Pharmaceutical and Biomedical Analysis* (2007), v. 45, p. 20-29. see 597.7(M+H)^+^/553.8(-CO2)/538.7(-CH2CO2H)/509.8(-2CO2)/494.8(-CH2CO2H,-CO2), 465.7((-3CO2). See data file C(H).raw for details.

******Figure 16.0. MS1 spectrum observed (Top) of 15^2^-Me rhodin g7** (M+H)^+^ *m/z* 625.26); R = 120k. **Middle**: calculated M^+.^; **Bottom:** calculated (M+H)^+^. See data file A(H).raw for details. See data file A(H).raw for details.

**Figure 16.1. Targeted HCD spectrum of 15^2^-Me rhodin g7** ((M+H)^+^ *m/z* 625.26); Q1 = 0.4 Th; R = 60k; CE 30_35_40. See data file C(H).raw for details.

**Figure 17.0. MS1 spectrum observed (Top) of 15^2^-Me chlorin e6** (M+H)^+^ *m/z* 611.28); R = 120k. **Middle**: calculated M^+.^; **Bottom**: calculated (M+H)^+^. See data file A(H).raw for details.

**Figure 17.1. Targeted HCD spectrum of 15^2^-Me chlorin e6** ((M+H)^+^ *m/z* 611.28); Q1 = 0.4 Th; R = 60k; CE 30_35_40. See data file C(H).raw for details.

**Figure 18.0. MS1 spectrum observed (Top) of 15^2^-Me isochlorin e4** (M+H)^+^ *m/z* 567.29); R = 120k. **Middle**: calculated M^+.^; **Bottom:** calculated (M+H)^+^. See data file A(H).raw for details.

**Figure 18.1. Targeted HCD spectrum of 15^2^-Me isochlorin e4: Top:** M^+.^ *m/z* 566.29; **Bottom**: (M+H)^+^ *m/z* 567.30; Q1 = 0.4 Th; R = 60k; CE 30_35_40. See data file C(H).raw for details.

**Figure 19.0. MS1 spectrum observed (Top) of Cu 15^2^-Me chlorin e6** (M^+.^ *m/z* 671.19, (M+H)^+^ *m/z* 672.20); R = 120k. **Middle**: calculated M^+.^; **Bottom:** calculated (M+H)^+^. See data file A(H).raw for details.

**Figure 19.1. Targeted HCD spectrum of Cu 15^2^-Me chlorin e6** (**Top**: M^+.^ *m/z* 671.19; **Bottom**: (M+H)^+^ *m/z* 672.20); Q1 = 0.4 Th; R = 60k; CE 30_35_40. See data file C(H).raw for details.

**Figure 20.0. MS1 spectrum observed (Top) of Cu isochlorin e4** (M^+.^ *m/z* 613.18, (M+H)^+^ *m/z* 614.19); R = 240k. **Middle**: calculated M^+.^; **Bottom:** calculated (M+H)^+^. See published lower resolution positive ESI-LCMS/MS work by Egner PA *et. al*. (2000) *Chem. Res Tox*, v. 13, p. 900-906.^5^ See data file B(H).raw for details.

**Figure 20.1. Targeted HCD spectrum of Cu isochlorin e4** (M^+.^ *m/z* 613.19); Q1 = 0.4 Th; R = 60k; CE 35_40_45. See published lower resolution positive ESI-LCMS/MS work by Egner *et. al*. 2000.^5^ See data file F(H).raw for details.

**Figure 20.2. Targeted HCD spectrum of Cu isochlorin e4:** **Top:** M^+.^ *m/z* 613.19; **Bottom:** (M+H)^+^ *m/z* 614.19; Q1 = 0.4 Th; R = 60k; CE 30_35_40. See published lower resolution positive ESI-LCMS/MS work by Egner *et. al*. 2000.^5^ See data file D(H).raw for details.

**Figure 20.3. Targeted HCD spectrum of Cu isochlorin e4** (M^+.^ *m/z* 613.19); Q1 = 0.4 Th; R = 60k; CE 30_35_40. See data file I(H).raw for details.

**Figure 21.0. MS1 spectrum observed (Top) of pyropheophorbide a** ((M+H)^+^ *m/z* 535.27); R = 120k. **Middle**: calculated M^+.^; **Bottom:** calculated (M+H)^+^. See data file A(H).raw for details.

**Figure 21.1. Targeted HCD spectrum of pyropheophorbide a** ((M+H)^+^ *m/z* 535.27); Q1 = 0.4 Th; R = 60k; CE 30_35_40. See data file E(H).raw for details.

**Figure 22.0. MS1 spectrum observed (Top) of Cu 15^2^-Me isochlorin e4 (**M^+.^ *m/z* 627.20); R = 30k. **Middle**: calculated M^+.^; **Bottom:** calculated (M+H)^+^. See data file H(H).raw for details.

**Figure 22.1. Targeted HCD spectrum of Cu 15^2^-Me isochlorin e4: Top:** M^+.^ *m/z* 627.20; **Bottom:** (M+H)^+^ *m/z* 628.21; R = 240k; Q1 = 0.4 Th; R = 60k; CE 35_40_45. See data file G(H).raw for details.

**C.) References for Supplementary Materials**

1. Mortensen, A.; Geppel, A., HPLC–MS analysis of the green food colorant sodium copper chlorophyllin. *Innovative food science & emerging technologies* **2007,** *8* (3), 419-425.

2. Aparicio-Ruiz, R.; Riedl, K. M.; Schwartz, S. J., Identification and Quantification of Metallo–Chlorophyll Complexes in Bright Green Table Olives by High-Performance Liquid Chromatrography–Mass Spectrometry Quadrupole/Time-of-Flight. *Journal of agricultural and food chemistry* **2011,** *59* (20), 11100-11108.

3. Delpino-Rius, A.; Cosovanu, D.; Eras, J.; Vilaró, F.; Balcells, M.; Canela-Garayoa, R., A fast and reliable ultrahigh-performance liquid chromatography method to assess the fate of chlorophylls in teas and processed vegetable foodstuff. *Journal of Chromatography A* **2018,** *1568*, 69-79.

4. Shioi, Y.; Watanabe, K.; Takamiya, K.-i., Enzymatic conversion of pheophorbide a to the precursor of pyropheophorbide a in leaves of Chenopodium album. *Plant and cell physiology* **1996,** *37* (8), 1143-1149.

5. Egner, P. A.; Stansbury, K. H.; Snyder, E. P.; Rogers, M. E.; Hintz, P. A.; Kensler, T. W., Identification and characterization of chlorin e4 ethyl ester in sera of individuals participating in the chlorophyllin chemoprevention trial. *Chemical Research in Toxicology* **2000,** *13* (9), 900-906.
